# Premature coronary artery disease and early stage chronic kidney disease

**DOI:** 10.1093/qjmed/hcx179

**Published:** 2017-09-18

**Authors:** A M Price, C J Ferro, M K Hayer, R P Steeds, N C Edwards, J N Townend

**Affiliations:** 1 Department of Nephrology, Birmingham Cardio-Renal Group, Institute of Cardiovascular Science, Queen Elizabeth Hospital Birmingham and University of Birmingham, Edgbaston, UK; 2 Department of Cardiology, Birmingham Cardio-Renal Group, Institute of Cardiovascular Science, Queen Elizabeth Hospital Birmingham and University of Birmingham, Edgbaston, UK

## Abstract

A 30 year old asymptomatic male with stage 3 chronic kidney disease (CKD) secondary to Focal Segmental Glomerulosclerosis was found to have features of CKD associated cardiomyopathy including left ventricular hypertrophy (LVH) and focal sub-endocardial scarring on cardiac magnetic resonance imaging. There was also a significantly raised CT coronary calcium score and evidence of non-flow limiting coronary artery disease (CAD) on a CT coronary angiogram. Early stage CKD is a major risk factor for cardiovascular risk causing myocardial hypertrophy and fibrosis and coronary artery atheroma. Cardiovascular risk begins to increase from an eGFR of around 75ml/min/1.73m^2^. The pathophysiology of cardiovascular disease in CKD is under investigation but to date, treatment options are limited. Blood pressure control and statins have the strongest supportive evidence.

## Introduction

The leading cause of death in patients with end-stage renal disease (ESRD) is cardiovascular disease.^1^ Sudden cardiac death, arrhythmia and congestive cardiac failure are more common than myocardial infarction (MI) suggesting that it is left ventricular disease (CKD-associated cardiomyopathy) rather than CAD that has the greater clinical impact.^2^ Cardiac imaging techniques have demonstrated that a large majority of patients with ESRD have structural and functional myocardial abnormalities and often evidence of accelerated CAD.^3^ Only more recently has it been appreciated that cardiovascular risk is also elevated in patients with early stage CKD and that early features of myocardial and coronary disease are common. Severe cardiovascular disease may be present even in young asymptomatic patients with early stage CKD.

## Case vignette

**Figure 1:**
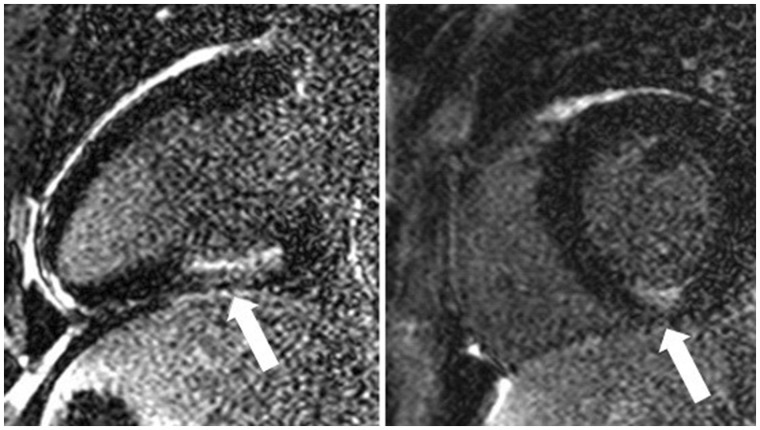
Focal sub-endocardial late gadolinium enhancement in the mid-inferior segment within the right coronary artery territory.

A 30-year-old non-smoking male was referred to hospital renal services due to a reduced estimated glomerular filtration rate (eGFR) of 57 ml/min/1.73 m^2^ and heavy proteinuria (albumin creatinine ratio 120.3 mg/mmol, albumin 40 g/l). There was no significant past medical history. He was hypertensive (164/72) but not obese. His total cholesterol was 5.3 mmol/l and triglycerides 1.10 mmol/l. He was started on losartan with initial adequate blood pressure (BP) control (129/84) but continued to have proteinuria. A renal biopsy showed focal segmental glomerulosclerosis (FSGS). A cardiac magnetic resonance (CMR) scan demonstrated concentric LVH with a maximal segmental wall thickness of 16 mm in the basal septum and an elevated left ventricular mass of 108 g/m^2^ (normal range for age and gender 40–97 g/m^2^). Systolic function was good with an ejection fraction of 61% and 3D global longitudinal strain (GLS) of −15.06%. There was however, hypokinesia of the mid-inferior and inferolateral segments without associated thinning. Following administration of intravenous gadolinium contrast there was focal sub-endocardial late enhancement in the basal and mid-inferior segment corresponding with the segments of hypokinesia (see Figure 1). These findings raised the possibility of MI due to CAD. A CT coronary angiogram (CTCA) showed moderate coronary calcification with an Agatston score of 112. There was evidence of mild diffuse coronary atheroma but no flow limiting stenosis within the main coronary arteries.

## Discussion

The high prevalence of cardiovascular disease in ESRD was identified in the 1970s early after the introduction of dialysis and was found to be the result of left ventricular disease (CKD-associated cardiomyopathy) and accelerated atherosclerosis.^2^^,^^4^ It is now the most common cause of death in this group with a prevalence of between 10 and 100 times control groups according to age.^5^^,^^6^ Only in recent years has the elevated cardiovascular risk of patients with early stage CKD been identified. There is a graded relationship between the degree of cardiovascular risk and eGFR so that the risk of early stage CKD is much lower than that of ESRD.^7^

In early stage CKD, left ventricular mass is frequently elevated and the reported prevalence of LVH by echocardiography is 32%.^2^ Studies using echocardiography and CMR in subjects with CKD stages 2 and 3 have shown that although LV ejection fraction is preserved compared to controls, other indices of systolic function including GLS (a marker of regional contractility) are abnormal.^8^ In an echocardiographic study of patients with stage 2 and 3 CKD Edwards *et al.*^9^ showed that both arterial and left ventricular end systolic elastances (stiffness) were increased with a preserved coupling ratio but at the expense of LV stiffness and haemodynamic instability, a pattern resembling heart failure with preserved ejection fraction. Diastolic filling velocities were reduced compared to controls while *E*/*e*′ was increased.^9^*E*/*e*′ is the ratio of mitral early filling maximal velocity to early myocardial relaxation velocity of the lateral mitral annulus and is an index of left atrial pressure. More recently, the application of T1 mapping CMR has allowed better characterization of the myocardium than with standard late gadolinium enhancement inversion recovery imaging. In a small cross-sectional study, Edwards *et al.*^8^ demonstrated an increase in extracellular volume (biomarker of diffuse interstitial fibrosis) with CKD compared to age and gender matched healthy controls and hypertensive subjects with normal renal function. This was correlated with a reduction in GLS.^8^ Furthermore, while gadolinium contrast agents are contraindicated in patients with ESRD due to previous reports of nephrogenic systemic fibrosis, native T1 mapping (without contrast) evades this issue.^10^ In a study of 28 haemodialysis patients compared to 33 age and gender-matched controls Rutherford *et al.*^10^ found significantly higher global and septal T1 values indicating diffuse fibrosis.

While novel techniques allow further insights into the structural changes seen with CKD-associated cardiomyopathy the pathophysiology of CKD-associated cardiomyopathy remains complex and multifactorial. To date, there is evidence supporting a disordered renin–angiotensin aldosterone system, altered calcium and phosphate metabolism (causing high levels of fibroblast growth factor 23 and parathyroid hormone) and hypertension.^11^ Effective treatment is still lacking. While angiotensin-converting enzyme (ACE) inhibition causes regression of left ventricular mass in those without CKD no such effect has been shown in CKD.^12^ ACE inhibition, however, still has a pivotal role in the management of CKD due to its antihypertensive and antiproteinuric effects which slow the progression of CKD.^13^ The addition of spironolactone to an ACE inhibitor or angiotensin receptor blocker (ARB) is promising. In a randomized blinded, placebo-controlled trial of patients with CKD stage 2 and 3, it reduced arterial stiffness and left ventricular mass with only rare associated hyperkalaemia.^14^

Early stage CKD is probably also an independent risk factor for CAD.^15^ In the ARIC study, the hazard ratios for atherosclerotic events were 1.38 and 1.16 for subjects with CKD stages 3–4 and 2, respectively.^16^ An analysis of Framingham data however, found early stage CKD to be strongly associated with coronary risk factors but it was not independently associated with cardiovascular events.^17^ In patients with established CAD, those with CKD tend to have more severe disease.^18^ CKD was associated with was an increase in mortality and re-infarction in almost 40, 000 patients recorded in databases of four acute coronary syndrome trials.^19^ There was an independent association of creatinine clearance with the hazard ratio for death at 180 days for both ST-elevation MI and non-ST elevation MI.^19^ The increased calcium score on CTCA in this patient is in keeping with previous studies which demonstrated calcium scores could be 8 times higher than those without CKD.^20^ The high prevalence and early onset of CAD in CKD is likely to be a combination of systemic inflammation, oxidative stress, hypertension, vascular calcification and disorders in bone metabolism.^21^

Management of CAD in CKD is challenging due to a lack of evidence.^22^ Despite the large studies supporting the use of aspirin, statins, ACE inhibition and beta blockade for those without CKD, patients with CKD are less likely to be started on such medications despite higher risks.^22^ Patients with CKD are often excluded from major cardiovascular studies.^22^ A recent meta-analysis of 50 studies found that antiplatelet agents reduced the risk of MI by 13% (a lower figure than the general population) but the risk of major bleeding was increased regardless of the of antiplatelet agent used or the stage of CKD.^23^ In addition Kim *et al.*^24^ found aspirin could have an adverse effect on renal function. Evidence for statins is a little clearer. Although randomized studies in dialysis patients found no reduction in death, non-fatal MI or stroke following the reduction of LDL the large Study of Heart and Renal Protection (SHARP), showed that the combination of simvastatin and ezetimibe caused a highly significant 17% reduction in atherosclerotic events.^25^^,^^26^ In SHARP the effects of statins on events was similar in both dialysis and non-dialysis patients but a meta-analysis showed that the reduction in major vascular events fell as the eGFR decreased, suggesting that subjects with early stage CKD gain most benefit.^25^ More recently a meta-analysis conducted by the Cholesterol Treatment Trialists Collaboration including 183 419 patients from 28 trials concluded that overall statin therapy reduced the first vascular event by over 20% in patients with CKD.^27^ The effect reduced as eGFR declined meaning there was little advantage for patients on dialysis.^27^

This young patient already has evidence of both CKD-associated cardiomyopathy with LVH and scarring and premature CAD with a possible previous MI. He is asymptomatic and none of these abnormalities were evident on routine clinical examination or ECG. There are no current guidelines on screening patients with early stage CKD for cardiac disease but this case should serve to remind all nephrologists of the need to be vigilant; subjects with early stage CKD are usually far more likely to die of premature cardiovascular disease (13-fold) than to progress to ESRD.^1^

## References

[1] Dalrymple LS, Katz R, Kestenbaum B, Shlipak MG, Sarnak MJ, Stehman-Breen C, et al Chronic kidney disease and the risk of end-stage renal disease versus death. J Gen Intern Med 2011; 26: 379–85. 20853156 10.1007/s11606-010-1511-x PMC3055978

[2] Di Lullo L, Gorini A, Russo D, Santoboni A, Ronco C. Left ventricular hypertrophy in chronic kidney disease patients: from pathophysiology to treatment. Cardiorenal Med 2015; 5: 254–66. 26648942 10.1159/000435838 PMC4662296

[3] Reddan DN, Szczech LA, Tuttle RH, Shaw LK, Jones RH, Schwab SJ, et al Chronic kidney disease, mortality, and treatment strategies among patients with clinically significant coronary artery disease. J Am Soc Nephrol 2003; 14: 2373–80. 12937316 10.1097/01.asn.0000083900.92829.f5

[4] Lindner A, Charra B, Sherrard DJ, Scribner BH. Accelerated atherosclerosis in prolonged maintenance hemodialysis. N Engl J Med 1974; 290: 697–701. 4813742 10.1056/NEJM197403282901301

[5] Thompson S, James M, Wiebe N, Hemmelgarn B, Manns B, Klarenbach S, et al Cause of death in patients with reduced kidney function. J Am Soc Nephrol 2015; 26: 2504–11. 25733525 10.1681/ASN.2014070714 PMC4587695

[6] Foley RN, Parfrey PS, Sarnak MJ. Clinical epidemiology of cardiovascular disease in chronic renal disease. Am J Kidney Dis 1998; 32: S112–S9. 9820470 10.1053/ajkd.1998.v32.pm9820470

[7] Chronic Kidney Disease Prognosis C. Association of estimated glomerular filtration rate and albuminuria with all-cause and cardiovascular mortality in general population cohorts: a collaborative meta-analysis. Lancet 2010; 375: 2073–81. 20483451 10.1016/S0140-6736(10)60674-5 PMC3993088

[8] Edwards NC, Moody WE, Yuan M, Hayer MK, Ferro CJ, Townend JN, et al Diffuse interstitial fibrosis and myocardial dysfunction in early chronic kidney disease. Am J Cardiol 1311; 115: 7. 10.1016/j.amjcard.2015.02.015 25769628

[9] Edwards NC, Ferro CJ, Townend JN, Steeds RP. Aortic distensibility and arterial-ventricular coupling in early chronic kidney disease: a pattern resembling heart failure with preserved ejection fraction. Heart 2008; 94: 1038. 18308865 10.1136/hrt.2007.137539

[10] Rutherford E, Talle MA, Mangion K, Bell E, Rauhalammi SM, Roditi G, et al Defining myocardial tissue abnormalities in end-stage renal failure with cardiac magnetic resonance imaging using native T1 mapping. Kidney Int 2016; 90: 845–52. 27503805 10.1016/j.kint.2016.06.014 PMC5035134

[11] Jardine A, McLaughlin K. Cardiovascular complications of renal disease. Heart 2001; 86: 459–66. 11559693 10.1136/heart.86.4.459 PMC1729948

[12] Mathew J, Sleight P, Lonn E, Johnstone D, Pogue J, Yi Q, et al Reduction of cardiovascular risk by regression of electrocardiographic markers of left ventricular hypertrophy by the angiotensin-converting enzyme inhibitor ramipril. Circulation 2001; 104: 1615–21. 11581138 10.1161/hc3901.096700

[13] Remuzzi G, Perico N, Macia M, Ruggenenti P. The role of renin-angiotensin-aldosterone system in the progression of chronic kidney disease. Kidney Int 2005; 68: S57–65. 10.1111/j.1523-1755.2005.09911.x 16336578

[14] Edwards NC, Steeds RP, Stewart PM, Ferro CJ, Townend JN. Effect of spironolactone on left ventricular mass and aortic stiffness in early-stage chronic kidney disease: a randomized controlled trial. J Am Coll Cardiol 2009; 54: 505–12. 19643310 10.1016/j.jacc.2009.03.066

[15] Anavekar NS, McMurray JJV, Velazquez EJ, Solomon SD, Kober L, Rouleau J-L, et al Relation between renal dysfunction and cardiovascular outcomes after myocardial infarction. N Engl J Med 2004; 351: 1285–95. 15385655 10.1056/NEJMoa041365

[16] Hui X, Matsushita K, Sang Y, Ballew SH, Fülöp T, Coresh J. CKD and cardiovascular disease in the Atherosclerosis Risk in Communities (ARIC) study: interactions with age, sex, and race. Am J Kidney Dis 2013; 62: 691–702. 23769137 10.1053/j.ajkd.2013.04.010 PMC3783539

[17] Culleton BF, Larson MG, Wilson PWF, Evans JC, Parfrey PS, Levy D. Cardiovascular disease and mortality in a community-based cohort with mild renal insufficiency. Kidney Int 1999; 56: 2214–9. 10594797 10.1046/j.1523-1755.1999.00773.x

[18] Chonchol M, Whittle J, Desbien A, Orner MB, Petersen LA, Kressin NR. Chronic kidney disease is associated with angiographic coronary artery disease. Am J Nephrol 2007; 28: 354–60. 18046083 10.1159/000111829

[19] Al Suwaidi J, Reddan DN, Williams K, Pieper KS, Harrington RA, Califf RM, et al Prognostic implications of abnormalities in renal function in patients with acute coronary syndromes. Circulation 2002; 106: 974–80. 12186803 10.1161/01.cir.0000027560.41358.b3

[20] Kramer H, Toto R, Peshock R, Cooper R, Victor R. Association between chronic kidney disease and coronary artery calcification: the Dallas Heart Study. J Am Soc Nephrol 2005; 16: 507–13. 15601745 10.1681/ASN.2004070610

[21] Moody WE, Edwards NC, Chue CD, Ferro CJ, Townend JN. Arterial disease in chronic kidney disease. Heart 2013; 99: 365–72. 23118349 10.1136/heartjnl-2012-302818

[22] Edwards NC, Steeds RP, Ferro CJ, Townend JN. The treatment of coronary artery disease in patients with chronic kidney disease. QJM 2006; 99: 723–36. 17040978 10.1093/qjmed/hcl101

[23] Razavian M, Di Micco L, Palmer SC, Craig JC, Perkovic V, Zoungas S, et al Antiplatelet agents for chronic kidney disease. Cochrane Database Syst Rev 2013; CD008834. DOI: 10.1002/14651858.CD008834.pub2. 10.1002/14651858.CD008834.pub2 23450589

[24] Kim AJ, Lim HJ, Ro H, Ko K-P, Han SY, Chang JH, et al Low-dose aspirin for prevention of cardiovascular disease in patients with chronic kidney disease. PLoS One 2014; 9: e104179. 25093403 10.1371/journal.pone.0104179 PMC4122498

[25] Group SC. Study of Heart and Renal Protection (SHARP): Randomized trial to assess the effects of lowering low-density lipoprotein cholesterol among 9, 438 patients with chronic kidney disease. Am Heart J 2010; 160: 785–94.e10. 21095263 10.1016/j.ahj.2010.08.012

[26] Wanner C, Krane V, März W, Olschewski M, Asmus HG, Krämer W, et al Randomized controlled trial on the efficacy and safety of atorvastatin in patients with type 2 diabetes on hemodialysis (4D study): demographic and baseline characteristics. Kidney Blood Press Res 2004; 27: 259–66. 15316128 10.1159/000080241

[27] Cholesterol Treatment Trialists Collaboration. Impact of renal function on the effects of LDL cholesterol lowering with statin-based regimens: a meta-analysis of individual participant data from 28 randomised trials. Lancet Diabetes Endocrinol 2016; 4: 829–39. 27477773 10.1016/S2213-8587(16)30156-5

